# Deep learning in plant phenotyping: the first ten years

**DOI:** 10.1016/j.plaphe.2025.100062

**Published:** 2025-08-20

**Authors:** Jordan Ubbens, Ian Stavness, Michael P. Pound, Wei Guo

**Affiliations:** a Aquatic and Crop Resource Development, National Research Council Canada, Canada; b Department of Computer Science, University of Saskatchewan, Canada; c Computer Vision Laboratory, School of Computer Science, University of Nottingham, UK; d Graduate School of Agricultural and Life Sciences, University of Tokyo, Japan

**Keywords:** Machine learning, Deep learning, Image analysis

## Abstract

As with many fields of science, plant science and agriculture have seen a rapid adoption of deep learning in recent years. The present moment is significant as it marks one decade since the first applications of deep learning began to appear in the literature on plant phenotyping. In this short time, a new research community was founded and new connections between computer vision and biology were established. In this letter, we reflect on this critical period of time from the inception of the field to where it stands today.

## 1 Introduction

Improvements in high-throughput imaging technologies in the 2000s, such as automated greenhouse photo booths, carts, and drones, saw plant scientists begin to generate large quantities of image data experimentally [1,2]. This explosion in the quantity of data created an urgent need for image analysis capability, in order to convert these images into biologically meaningful measurements of plant phenotypes. Image analysis quickly stepped in to meet this need, providing important measurements from images such as geometry, color, and reflectance [3,4]. Despite much progress, accurate phenotyping pipelines based on traditional image analysis techniques were often costly to create, with automation quickly becoming a bottleneck to progress [5]. Just as progress appeared to slow, deep learning emerged as the new dominant technique in the computer vision literature [6]. Where traditional image analysis techniques required careful handling of many varied difficult cases, deep learning re-framed the problem to focus more on useful and varied datasets. This rapidly evolving field promised to revolutionize image-based plant phenotyping, and it was with optimism that the first applications began to appear in the mid-2010s.

**Fig. 1:**
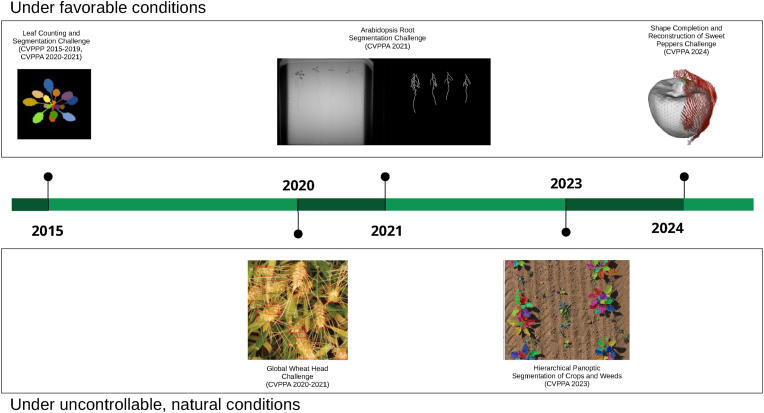
An example of how plant phenotyping tasks have changed over time, exemplified by the evolution in challenge datasets launched at the CVPPP/CVPPA series of conferences from 2015 to 2024.

With the benefit of ten years of hindsight, it is evident now that the field of deep learning has had wide reaching impact on how plant phenotyping is performed, and also that deep learning applications have changed in response to end-user needs as well as data challenges. Initially focused on hyper-specialized, proof-of-concept results, the field shifted over the years to focus on broader challenges such as different types of imaging data (Fig. 1) and the generalization difficulties associated with small and homogeneous plant image datasets. Very recently, we have seen the field align with a movement in the broader computer vision field towards large foundation models which are trained on general imagery via self-supervision. With this shift of focus and priorities over the last decade, there is now an opportunity to reflect and readjust.

Many review articles are available that provide good overviews of the current body of deep learning in plant phenotyping literature [[7], [8], [9], [10]]. Rather than providing an exhaustive list of techniques and applications, in this paper, we instead aim to zoom out and offer a perspective of this nascent field starting from the beginning, showing how it has evolved in its aims and expectations over its first decade. For each topic, we highlight the earliest results for each technique or application to discuss how the idea was introduced to the field. Finally, we use the lessons learned throughout the history of deep learning in plant phenotyping to make recommendations for steering the future of the field towards new opportunities and away from historically unproductive directions.

## 2 2015–2017: the first specialist models

### 2.1 Proofs of concept

The first applications of deep learning to plant phenotyping targeted plant disease classification [11,12] and organ counting [[13], [14], [15]]. The early focus on these two tasks was due in part to the availability of seminal datasets including PlantVillage [16] and the Leaf Counting Challenge dataset [17]. These studies served as proofs-of-concept for deep learning in plant phenotyping – they demonstrated what was possible using these new methods, which is distinct from what was previously possible using standard image processing approaches. Now, in addition to the geometric and reflectance characteristics provided by image processing, users could extract higher-level phenotypes by learning from annotated training data. In some cases, these new machine-measured phenotypes had high agronomic importance, such as the number of heads, tassels, or spikelets. Performance on these early problems was encouraging, with methods based on deep neural networks typically surpassing previous results which used classical image processing techniques [13].

### 2.2 A research community gains traction

The emergence of standardized public benchmark datasets and competitions spurred a rapid increase in interest from the computer vision community and the field attracted a large influx of researchers during these initial years as a result [18]. While initially small, the deep learning in plant phenotyping community exploded in the late 2010s, with new meetings, collaborations, and the rapid progression of ideas. International collaborations flourished, and the relatively small plant phenomics community, composed mainly of biologists, came together with computer scientists and their own computer vision communities. New workshops focused on deep learning in plant phenotyping such as Computer Vision in Plant Phenotyping and Agriculture (CVPPA, formerly CVPPP), Machine Learning for Cyber-Agricultural Systems (MLCAS), and Agriculture-Vision were hosted at established vision meetings such as the International Conference on Computer Vision (ICCV) and Computer Vision and Pattern Recognition (CVPR). Early iterations of these workshops had 20-50 attendees, and now commonly host over 100 attendees each year. The field continues to expand to this day, with more meetings and community datasets being announced regularly.

## 3 2018–2022: new modalities and new challenges

### 3.1 The field explores new sensors and applications

Plant science and agronomy have been early adopters of a diverse array of different sensors, as plants can be measured and quantified at every scale from the microscopic to the field level. With this diversity in sensors comes a diversity of different data types and applications, presenting opportunities for applying deep learning. Although the initial demonstrations focused on RGB imagery, soon other applications were explored using novel types of data. These applications often borrowed heavily from the existing computer vision literature, translating previous research to unlock new capabilities in agriculture.

While early applications focused on imagery taken with standard consumer cameras in the visible light spectrum, this type of sensor can be restrictive for plant phenotyping. Hyperspectral imagery, the measurement of many different wavelengths including those outside the visible spectrum, has long been used to assess reflectance characteristics which are not visible to the human eye. This is common for both remote sensing applications, where aerial or satellite imagery is used to characterize large areas such as crop fields, as well as proximal imaging of single leaves or other plant organs [19]. Deep learning has been applied to hyperspectral data routinely, and so its application to plant phenotyping in the hyperspectral domain was quickly investigated. Early attempts focused on the macro scale, predicting disease at the field level [20] and cold stress at a lower scale [21]. As with RGB imagery, authors often reported that deep learning offered increased performance over classical methods in hyperspectral tasks.

Another prominent type of data which is also broadly applicable to plant phenotyping is volumetric data. Unlike standard imaging which provides only a two-dimensional projection of the scene, volumetric data provides density in all three dimensions, accounting for the full three dimensional nature of the scene. 3D input data is useful for many purposes, as it allows one to handle self-occlusion and reason about traits which are only observable in 3D, such as leaf angle. 3D data is especially relevant in robotics applications, since robots are required to navigate in real space. Robust volumetric representations of a scene can therefore enable applications such as automated fruit harvesting from fruit trees. Some early work applying deep learning to 3D plant data involved the volumetric measurement of plants using next-best view planning [22], as well as 3D organ segmentation based on a dataset of plants obtained via laser scanning [23]. An alternative to volumetric data is depth cameras, which capture depth information in a scene from only a single viewpoint. In plant phenotyping, consumer-grade depth cameras were used to improve the quality of organ segmentation using deep learning [24]. Light Detection and Ranging, or LiDAR, is another sensor which can provide accurate depth information from either a static viewpoint, or mounted on a moving terrestrial or aerial vehicle [25]. Experiments have shown that deep learning shows strong performance for plant organ segmentation when provided with LiDAR point clouds [26].

Recent advances in 3D capture utilize radiance field rendering methods, which are optimized to perform novel viewpoint synthesis – generating a new viewpoint which was not captured beforehand. Neural Radiance Fields (NeRF) [27] train neural networks to learn to represent the light field around the scene. 3D Gaussian Splatting (3DGS) [28] optimizes an explicit representation of the 3D scene being captured by adjusting the position, shape, and radiance of many small 3D gaussians. While focused on view synthesis, these approaches have the added effect of building a detailed representation of a scene. Radiance field and gaussian splatting methods are applicable to plant phenotyping, providing detailed 3D understanding without the need to comprehensively scan the plant to construct a point cloud, voxel reconstruction, or mesh. Both NeRF and 3DGS methods have the potential to dramatically increase the speed, ease, and accuracy of 3D plant capture, but still require many images from views surrounding the plant and also typically require the plant to be stationary, which may be challenging for windy field conditions. NeRF based 3D plant reconstructions have been explored in plant phenotyping in both controlled environments [29], as well as outdoor field environments [[30], [31], [32], [33]]. 3DGS based plant reconstructions have also been demonstrated in preliminary studies for cotton [34], wheat [35], and other plant species [36]. Future studies are needed to elucidate the trade-offs between NeRF and 3DGS approaches for 3D plant reconstruction, and the development of robust analysis pipelines to extract phenotypic information from 3D radiance fields will be needed for wide-spread and routine use of volumetric data in plant phenotyping studies.

### 3.2 Generalizing to out-of-distribution data a problem

Following the introduction of the first models for plant phenotyping tasks, many authors turned their attention to the practical realities of these models when used in production. The most major factor limiting practical applicability was found to be domain shift: the model's loss of generalization capability when presented with data outside of the distribution used for training, i.e. the user's own private data [[37], [38], [39]]. This would become an intense area of investigation in the following years, with different authors taking varied approaches to the problem. Throughout and following this period of time, authors would continue to explore the problem of domain shift.

As with many application areas in machine learning, data scarcity is a pervasive issue in deep learning for plant phenotyping. Although field and indoor plant image datasets can be extremely large with respect to the raw quantity of data, they often fail to represent a comprehensive range of variation when it comes to biological factors such as genotype and maturity, as well as imaging factors such as perspective, background, lighting, imaging modality, resolution, and camera calibration. During this time, the community saw a proliferation of new plant imaging datasets being released publicly, which in turn, helped to spur more interest in plant problems within the deep learning community. However, few multi-domain and high-diversity datasets were available. In one attempt to address the lack of multi-domain training data, the Global Wheat Dataset initiative was launched to compile a large and diverse image dataset for wheat head counting [40,41]. The Global Wheat Head Detection (GWHD) dataset was designed to advance inter-domain generalization by including sub-datasets from a diversity of different geographic domains in both the training and testing sets. GWHD was successful in capturing the attention of deep learning researchers through public competitions [42]. The winning models from these competitions, though based on standard architectures, demonstrated the potential to replace manual field counting methods, particularly under well-defined conditions. The lessons learned from those competitions indicate the importance of continuous refinement in model architecture and data processing techniques. Additionally, GWHD became a sub-dataset within a larger meta-dataset that has become a benchmark for studying in-the-wild distribution shift problems [43,44].

It can be costly to obtain sufficient variation in natural image data, and so one alternative has been the generation of synthetic image data. Synthetic data pipelines allow the user to specify by hand the variation which should be represented in the data. There are several ways in which synthetic data can be generated, such as structural-functional plant models [38], generative neural networks [37,45], and 2D and 3D compositing [46,47]. Some of these techniques showed some limited improvement in generalization, but often at a significant cost in time and effort. Although many functional-structural plant models already exist for common plant species of interest, they need to be modified by hand to make them appropriate for the task of generating training data by modulating model parameters and rendering settings until they are a significant visual match for the target dataset. This process can reduce the time and effort saved by using synthetic data versus simply collecting and annotating additional real-world data. Meanwhile, attempts to generate synthetic training data using generative networks have had mixed results, typically providing small or insignificant performance advantages. This has been recapitulated in other fields, and may be due to different supports between the real and synthetic data, or the inability of the generative model to accurately reason about phenotypes outside of those represented in the data it was trained on. Compositing techniques, on the other hand, are an effective way to inject into the training data prior knowledge about the objects and the different contexts in which they could feasibly appear. However, these techniques are limited in a different way, by typically only representing objects which already exist elsewhere in the data. This makes compositing a form of augmentation more like image-based augmentations such as rotation and flipping than truly synthetic generation such as via plant models or generative networks. Despite the only modest use of synthetic data in phenotyping to-date, there are reasons to be optimistic for this field over the next few years. Very recent advancements in image generation [48,49] and efficient fine tuning [50] and control of models [51] have had a profound impact on other fields such as the generation of artistic images. These technologies have the potential to improve future synthetic image generation efforts in plant phenotyping, and early work is yielding promising results [52]. View rendering techniques such as 3DGS [28] also facilitate highly realistic compositing of objects, a potentially useful property for plant images.

Another approach to the domain shift problem adapted from the broader literature is domain adaptation. These techniques are usually classified as either unsupervised and semi-supervised domain adaptation. In unsupervised domain adaptation, annotations from an annotated source dataset are transferred to a target dataset which does not provide any annotations, while in semi-supervised domain adaptation, the target dataset is equipped with a small number of annotations. Domain adaptation has been explored in organ counting problems [39,53]. Unsupervised domain adaptation is in some respect the gold standard, as it does not require any annotation effort on the part of the end-user. However, without annotations, domain adaptation techniques are largely constrained by the similarity between the source and target datasets. For example [39], found that their domain adaptation method saw an uplift in the coefficient of determination for an out-of-domain leaf counting problem from 0.09 to 0.34 for an intra-species task, and from 0.01 to 0.38 for an inter-species task. This is a substantial improvement, but it also shows how the performance ceiling for unsupervised domain adaptation may be too low for practical use in some cases. Other authors have combined the techniques of 3D modelling and domain adaptation [54,55]. This can help to alleviate the burden of manually aligning the appearance of the 3D renders with the appearance of the target dataset. However, it does not completely obviate the gap between the synthetic and real data and requires additional effort for tuning both the 3D modelling process as well as the domain adaptation process.

## 4 2023 – present: leveraging strong generalist vision models

### 4.1 Foundation models for vision enter plant phenotyping

Although somewhat insulated from the more data-rich application areas, plant phenotyping has been affected recently by rapid progress elsewhere in computer vision. With the recent meteoric rise of large language models (LLMs) in natural language processing, much effort has been expended by the wider computer vision community to develop foundation models for vision as well [56,57]. These models are typified by being extremely large in their number of parameters, and being trained on extremely large datasets, usually via self-supervision. The result is strong visual representations which can be used for tasks such as annotation-free object segmentation in natural images. Recent efforts have explored the usefulness of self-supervised learning and foundation models for vision with respect to existing plant phenotyping tasks [[58], [59], [60], [61], [62]].

Early results show that these foundation models do show some capacity for understanding plants and plant parts. However, the authors are largely in consensus that, although there is potential in these models for plant phenotyping tasks, they are not appropriate for the task “out-of-the-box”. The authors are only able to achieve comparable results to existing methods by layering the outputs of the foundation model with further processing of the outputs based on hand-made heuristics, such as thresholding by green pixels, object shape, or other factors [61,62]. Even after fine-tuning on plant data, these models often underperformed existing purpose-built solutions from the literature [60]. However, given their strong performance in other domains, there is optimism for the future of vision pipelines in plant phenotyping based on foundation models as a strong pre-trained visual backbone [59].

### 4.2 Visual prompting provides an alternative for homogeneous datasets

**Fig. 2:**
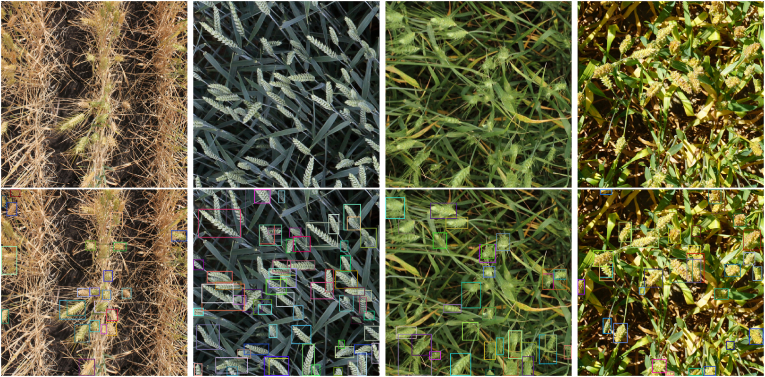
An example of visual prompting using T-Rex2 [63] with some example images from the Global Wheat dataset [40]. Input images (top) were provided with a visual prompt in the form of a single point annotation per image, resulting in detections (bottom).

Since plant phenotyping datasets tend to be homogeneous in practice, trained models tend to do poorly on end-users’ dataset due to domain shift. However, this homogeneity can be an advantage in another context. Recent models have been developed which allow users to provide input in the form of a visual prompt, which is an annotation of an object of interest. Since plant datasets are homogeneous, a single visual prompt of a spike, tassel, or lesion on one image may suffice to perform inference on an entire dataset, because successive images do not vary much in their content. Unlike classical annotation techniques such as active learning, these models are trained to do the detection task given a visual prompt and do not require re-training when provided with the prompt. Fig. 2 shows an example of wheat head detection using the T-Rex2 visual model [63]. An example from each of four domains from the Global Wheat dataset are shown. On inspection, a single visual prompt for each image does seem to result in sensible predictions for wheat head detection. There is evidence of under-detection in each case, in particular for the first domain (left), but the model seems to be effective at detecting the densely overlapping wheat heads without many false positives or duplicate detections. This is an encouraging result given the difficult nature of wheat head detection, especially given a single annotation.

## 5 Conclusions

### 5.1 Has the plant science community benefited?

The deep learning in plant phenotyping community provides a technological support system for plant scientists, plant breeders, agronomists, and other professionals. The purpose of this research should be to enable data collection from images which is faster, more accurate, and requires less human time and effort than before. It is difficult to quantify how much deep learning efforts have resulted in useful tools for these end users so far – however, some authors have demonstrated their use in the literature [64]. described how an object detection approach was used for maize kernel quantification. Despite an estimated annotation requirement of approximately 100 ​h, the authors found utility in the approach as it offered high throughput and good accuracy. The authors noted problems with domain shift owing to the use of different cameras [65]. used RootNav 2.0 [66] to automate the analysis of 2D root systems. The authors noted that the analysis time of approximately 5 ​s per image enables the screening of hundreds of lines.

### 5.2 Course correction: where do we go from here?

Given the substantial public investment in deep learning in plant phenotyping research over the last decade, it is sensible to pause and consider how the field can position itself to have the maximum possible practical impact going forward. Ideally, the most useful output would be models for common phenotyping tasks which can be run routinely, generalize easily to end-users’ datasets, and can be run on commodity compute hardware without any further work or specialized skills. However, this goal still seems to be largely out of reach.

Based on collective experience, there are a few promising research directions. Domain shift seems to remain one of the most pressing problems facing the field. As we have seen, the issue with domain shift first described around 2017 continues to be largely unsolved – that is, most specialist plant phenotyping models described in the literature will perform poorly unless they are re-trained on annotated training data which matches the target dataset where inference is to be performed. Doing so is impractical on two fronts: end users typically lack the technical knowledge required to effectively re-train a model, and the effort required to annotate a significant amount of training data often negates the automation advantages of using image-based phenotyping. Another avenue to impact is to increase the ease with which software systems can be re-trained on target data. This can be done by engineering software with user-friendly interfaces which allow models to be re-trained without the need for specific technical skills. This can alleviate the burden from the user of learning the minutia of training neural networks, but it does not address the need for annotation. In the root domain, RootNav 2 [66] provides a simple command line interface by which models can be retrained to new data. However this tool still requires limited expertise to obtain and configure the relevant libraries and software, as well as a training dataset comprising perhaps 100 images. RootPainter [67] combines annotation and training into a single tool, with a graphical user interface in which users can paint foreground areas by hand which inform the network training. Similarly, AgriCounter [68] provides a user-friendly interface for re-annotating and re-training detection models for seedling detection in aerial field images.

The field of deep learning in plant phenotyping has reached a pivotal intersection with the broader computer vision field. Large foundation models have been trained for general visual tasks, providing impressive performance across tasks such as object detection and segmentation on natural images. A natural path forward appears to be leveraging these large models for the benefit of plant phenotyping applications, although early investigations show that they have not yet completely obviated the need for purpose-built specialist models for plant phenotyping tasks, even when fine-tuned on annotated plant data [[59], [60], [61], [62]]. Conversely, bespoke models trained for plant phenotyping tasks still tend to be affected by the problem of domain shift, similarly limiting their usefulness. At the current point in time, it appears that specialist models are not general enough, while generalist models are not specialized enough. Despite this, the continued development of adaptation techniques for foundation models has the potential to provide breakthroughs in generalization capability for plant phenotyping in the future.

One research direction which should be avoided is the hand-engineering of neural network architectures for a specific dataset, unless there is a specific demonstrated need. State-of-the-art architectures for visual tasks such as detection and segmentation tend to translate well to plant phenotyping tasks and there is little reason to believe that a task like localizing plant organs requires a different approach to any other detection task, such as crowd counting or autonomous driving. There is also considerable prior work across phenotyping domains that can be leveraged to good effect. Authors often contrive to develop a new network architecture for a specific application by tweaking the network until it performs slightly better than a given baseline on the test set, effectively overfitting the architecture to the test data. Indeed, we observe that for publications such as [66], many citing works are those that are also contributing new and different deep learning techniques to the same problem. Machine learning practitioners should reflect on whether a new technique is required, or whether an existing and often open source tool can be adapted or improved for the community. This is not to say that a “one size fits all” approach is universal in computer vision but merely that, across all possible maize tassel detection datasets for example, state-of-the-art architectures or an existing tool will likely perform well with minimal redevelopment. Researchers are then able to take on the substantive remaining challenges in AI for plant phenotyping.

It is a unique privilege to be present at the inception of an entirely new application area in science. The first decade of deep learning in plant phenotyping saw rapid growth in the field, from the first demonstrations of deep convolutional neural networks for plant phenotyping tasks to recent experiments with state-of-the-art foundation models. Although the field is still challenged by some of the same problems from early on, such as domain shift, it is impressive to see the breadth of research that the community has undertaken during this time. From integrating 3D plant models and neural rendering, to all manners of imaging such as rhizoboxes, microscopy, automated greenhouses, drones, and gantries, there is no corner of plant phenomics which has not been advanced by deep learning. With some careful attention paid to where efforts are focused, the field is well positioned to deliver transformational results in the decade from 2025 to 2035.

## Author contributions

Jordan Ubbens: Conceptualization, Investigation, Writing-Original draft preparation, Writing-Review & Editing. Ian Stavness: Writing-Original draft preparation, Writing-Review & Editing. Michael Pound: Writing-Original draft preparation, Writing-Review & Editing. Wei Guo: Writing-Original draft preparation, Writing-Review & Editing.

## Declaration of competing interest

The authors declare that they have no known competing financial interests or personal relationships that could have appeared to influence the work reported in this paper.
